# Cell biology and genetics of minimal change disease

**DOI:** 10.12688/f1000research.7300.1

**Published:** 2016-03-30

**Authors:** Moin A. Saleem, Yasuko Kobayashi

**Affiliations:** 1 Paediatric Renal Medicine, University of Bristol, Bristol, UK; 2 Children's Renal Unit, Bristol Royal Hospital for Children, Bristol, UK; 3 Department of Pediatrics, Gunma University Graduate School of Medicine, Maebashi, Gunma, Japan

**Keywords:** Minimal change disease, steroid-sensitive nephrotic syndrome, focal segmental glomerulosclerosis, steroid-resistant nephrotic syndrome, circulating factor, permeability, podocyte

## Abstract

Minimal change disease (MCD) is an important cause of nephrotic syndrome and is characterized by massive proteinuria and hypoalbuminemia, resulting in edema and hypercholesterolemia. The podocyte plays a key role in filtration and its disruption results in a dramatic loss of function leading to proteinuria. Immunologic disturbance has been suggested in the pathogenesis of MCD. Because of its clinical features, such as recurrent relapse/remission course, steroid response in most patients, and rare familial cases, a genetic defect has been thought to be less likely in MCD. Recent progress in whole-exome sequencing reveals pathogenic mutations in familial cases in steroid-sensitive nephrotic syndrome (SSNS) and sheds light on possible mechanisms and key molecules in podocytes in MCD. On the other hand, in the majority of cases, the existence of circulating permeability factors has been implicated along with T lymphocyte dysfunction. Observations of benefit with rituximab added B cell involvement to the disease. Animal models are unsatisfactory, and the humanized mouse may be a good model that well reflects MCD pathophysiology to investigate suggested “T cell dysfunction” directly related to podocytes *in vivo*. Several candidate circulating factors and their effects on podocytes have been proposed but are still not sufficient to explain whole mechanisms and clinical features in MCD. Another circulating factor disease is focal segmental glomerulosclerosis (FSGS), and it is not clear if this is a distinct entity, or on the same spectrum, implicating the same circulating factor(s). These patients are mostly steroid resistant and often have a rapid relapse after transplantation. In clinical practice, predicting relapse or disease activity and response to steroids is important and is an area where novel biomarkers can be developed based on our growing knowledge of podocyte signaling pathways. In this review, we discuss recent findings in genetics and podocyte biology in MCD.

## Introduction

Minimal change disease (MCD) is characterized by massive proteinuria without histological evidence of immune-mediated damage in the glomeruli. The glomerular podocyte plays a key role in filtration and its loss of function results in loss of protein, mainly albumin or smaller proteins, into the urine with high selectivity ^1^. Proteinuria in MCD is typically reversible with corticosteroid therapy ^2^. T cell dysfunction and circulating factors have long been implicated as a cause of the podocyte dysfunction in MCD ^3^, but their nature still remains to be elucidated.

Recent progress in genetics and cell biology has revealed the molecular mechanisms of dysfunction in podocytes ^4^. These findings give us clues to focus on target molecules on the podocyte to deduce what those circulating factors may be. At the same time, we can utilize those molecules as biomarkers not only as a diagnostic tool but also in predicting the disease activity or prognosis. This allows us to administer more accurate and precise treatment to patients with MCD while minimizing side effects caused by drugs.

Alongside MCD as one circulating factor disease is a subset of patients with the histological finding of focal segmental glomerulosclerosis (FSGS). These patients are mostly steroid resistant, and therefore the term steroid-resistant nephrotic syndrome (SRNS) is also used here. These patients often have a rapid relapse after transplantation, indicating another circulating factor disease. It is likely that at least a subset of patients with MCD progress to FSGS/SRNS, with a consistent circulating factor in both. The most compelling evidence for this is the observation that patients with initial steroid sensitivity (assumed to be MCD at that stage) who over subsequent years develop steroid resistance/FSGS, and renal failure, have a 90% chance of post-transplant disease recurrence – the archetypal manifestation of circulating factor disease ^5^. In this article, known pathogenesis and mechanisms underlying MCD are reviewed.

## Clinical features of MCD

MCD is the most common cause of nephrotic syndrome in children ^6^ and around 15–20% of cases in adults ^7^, and is characterized by massive proteinuria and hypoalbuminemia, resulting in edema and hypercholesterolemia. Histological findings of the disease in glomeruli are typically normal by light microscopy and only electron microscopy shows effacement of podocyte foot processes without electron-dense immune deposits ^8^. These manifestations are typically reversible with the use of corticosteroid therapy in steroid-sensitive nephrotic syndrome (SSNS), so that progressive loss of renal function is rare.

The incidence of MCD in childhood is twofold higher in boys, with a prevalence that is inversely proportional to age. Relapse occurs in 50–80% of patients, and recurrent relapse tends to lessen after adolescence ^9^. A genetic defect cannot explain these phenomena in MCD.

## Genetics in MCD

### Pathogenic mutations in MCD

Familial cases are rather rare in MCD, therefore the genetic background of SSNS is largely unknown, while 23.6% of SRNS cases ^10^ and 29.5% of familial SRNS cases ^11^ are caused by gene mutation. More than 24 genes are currently known to be pathogenic in SRNS ^12^ and have already been clinically utilized in practice in SRNS cases.

Recently, using whole-exome sequencing, several mutations were found in pedigrees with SSNS, which shed light on new mechanisms of podocyte disruption in MCD. Epithelial membrane protein 2 (EMP2) is known to regulate the amount of caveolin-1 ^13^, which contributes to endocytosis and the transcytosis of cholesterol and albumin ^14^. Lipopolysaccharide (LPS)-induced caveolin-1 phosphorylation was reported to lead to the increase of transcellular permeability ^15^.

More recently, recessive mutations in the *KANK* gene were identified in familial SSNS and in sporadic SRNS cases ^16^. Kidney ankyrin repeat-containing protein (KANK) family proteins have essential roles in podocyte/nephrocyte function and regulate Rho GTPase activity. KANK2 interacted with Rho GDP dissociation inhibitor alpha (ARHGDIA), a known regulator of Rho GTPases in podocytes found to be dysfunctional in SRNS ^17^. Knockdown of KANK2 in cultured podocytes increased active GTP-bound RHOA and decreased migration.

In these cases, we might have evidence of overlap of SSNS and SRNS. Also, it is important to know the mechanisms of how corticosteroid and immunosuppressants have their effect on nephrotic syndrome caused by single gene mutation.

## T cell dysfunction in MCD

T cell dysfunction has long been postulated and many types of cytokines have been investigated. One of the difficulties in examining a hypothesis that immunological disruption underlies MCD in the laboratory is the lack of an animal model that reflects the pathophysiological mechanism. Haddad *et al.* employed unique methods and established a nephrotic syndrome model by injecting CD34+ peripheral stem cells obtained from FSGS and MCD patients ^18^ rather than injecting the supernatant of T cells or peripheral blood mononuclear cells (PBMCs) obtained from the patients ^19^. The injected cells successfully induced the engraftment of human CD45 leukocytes in the thymus, and only the injection of CD34+ stem cells from patients induced albuminuria. Interestingly, stem-cell-injected mice did not have CD3+ mature T cells, suggesting that the cells responsible for the pathogenesis of idiopathic nephrotic syndrome are more likely to be immature differentiating cells rather than mature peripheral T cells. Naïve T cells (Th0s) have been focused on to investigate the difference in DNA methylation in MCD patients ^20^. The change in DNA methylation patterns from remission to relapse occurs predominantly in Th0s. Epigenetic involvement in the pathogenesis of minimal change nephrotic syndrome in T cells has also been suggested in a report showing that nuclear factor related to kappaB binding protein (NFRKB) was highly expressed in the nuclear compartment in T lymphocytes of MCD patients during relapse and that NFRKB promotes hypomethylation of genomic DNA in HEK cells transfected with NFRKB expression plasmid ^21^.

**Figure 1.**
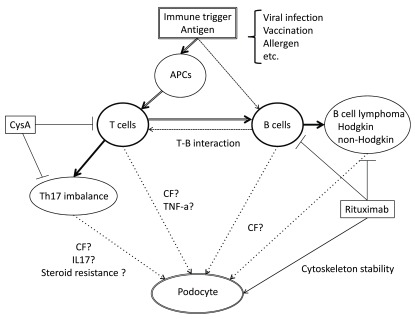
Scheme of lymphocyte dysfunction and circulating factors in minimal change disease (MCD). By immune trigger such as viral infection, vaccination, and exposure to allergen, antigen-presenting cells and memory B cells present antigen to T lymphocyte. These cells are stimulated to secrete circulating factors in MCD. Rituximab depletes B cells and induces remission; on the other hand, rituximab has an effect on cytoskeleton stability of podocytes and blocks albumin permeability. Th17 skew in MCD may cause steroid resistance and cyclosporine A selectively attenuates Th17. Abbreviations: APCs, antigen presenting cells; CysA, cyclosporine A; CF, circulating factor; IL17, interleukin 17; Th17, helper T subset 17; TNF-a, tumor necrosis factor alpha.

Another T cell dysfunction is a Th17 skew in MCD ^22, 23^ ( Figure 1). Patients with SSNS demonstrated after corticosteroid treatment that the Th17/regulatory T cell (Treg) balance returned to normal ^24^. More recently, it was reported that Th17 cells are strong candidate drivers for steroid resistance in immune diseases and have selective attenuation by cyclosporine A ^25^. This could be utilized to predict steroid response in early stages of nephrotic syndrome onset by testing peripheral Th17 levels.

## Rituximab and B cell dysfunction

A potential close pathophysiological relationship between MCD and chronic lymphoid neoplasms such as Hodgkin and non-Hodgkin lymphoma has been known since the 1950s, supporting a potential role for B cells in the pathogenesis of MCD ( Figure 1). A significant association of HLA-DQA1 (a major histocompatibility complex [MHC] class II) missense coding variants with SSNS recently suggested the possible role of an immune response and the implication of B cells in the pathogenesis of MCD ^26^.

Though the accurate mechanism by which rituximab, a monoclonal antibody against CD20, induces remission in MCD patients remains uncertain, recent observations of the effect of rituximab on complicated refractory SSNS ^27– 29^ suggests a pathophysiological role for B cells in MCD ^30, 31^ ( Figure 1). B cell depletion by rituximab resets and suppresses B cell and T cell interactions and keeps the Th17/Treg balance normal, which may lead to sustainable remission ^32, 33^. On the other hand, a direct role for rituximab on podocyte cytoskeleton stabilization was suggested: rituximab prevents disruption of the actin cytoskeleton in cultured normal human podocytes that have been exposed to FSGS patient sera in a sphingomyelin phosphodiesterase acid-like 3b-dependent manner ^34^.

## Circulating factors and podocyte cell biology in MCD

A direct test of “circulating factor” activity is to expose human podocytes in culture to active human disease plasma and examine the direct cellular effects on this target cell. It has been shown using this method that nephrotic plasma alters slit diaphragm-dependent signaling and translocates nephrin, podocin, and CD2-associated protein in cultured human podocytes ^35^. This indicated that there is a certain factor increasing or missing in MCD disease plasma.

## Hemopexin

Hemopexin (Hpx) is a circulating plasma protease that is synthesized in the liver. The active isoform of Hpx is increased in children with MCD ^36^. *In vitro*, podocytes showed dramatic reorganization of actin with loss of stress fibers after Hpx treatment ^37^. The Hpx effect on actin is dependent on nephrin followed by RhoA activation and protein kinase B phosphorylation in the downstream intracellular signaling pathway. The effects were reversible and were inhibited by pre-incubation with healthy human plasma or serine protease inhibitors. Though the mechanisms of Hpx activation in the disease are unclear, LPS and tumor necrosis factor (TNF)-α are indicated as possible triggers to activate Hpx in MCD ^38^.

## PAR1 signaling axis and VASPp, or suPAR

Because it has serine protease activity ^39^, Hpx may act via the family of protease-activated receptors. There are also matrix metalloproteinases among those proteins that have Hpx homology domains. Recent studies investigated the possibility of a matrix metalloproteinase–protease-activated receptor 1 (PAR1) signaling axis ^40^. It was recently reported that proteases present in nephrotic plasma obtained from patients with FSGS can activate PAR1, leading to the podocin-dependent phosphorylation of the actin-associated protein vasodilator-stimulated phosphoprotein (VASP) in human podocytes and increased cell migration, suggesting a novel role for proteases and PARs in the pathogenesis of FSGS ^41, 42^. Although the exact component(s) of FSGS plasma that causes this response remains unknown, the soluble urokinase plasminogen activator receptor (suPAR) has been identified as a potential circulating factor in FSGS via activation of β3 integrin in podocytes and promotes cell motility ^43– 45^. However, correlation of disease activity with suPAR levels has been inconsistent in subsequent reports ^46, 47^. Urinary suPAR was increased in MCD relapse, but it is thought it may simply be a surrogate for proteinuria ^48^. These factors are found in FSGS but are potentially also relevant to MCD; this needs experimental verification.

### CD80

CD80 (B7-1) is a T cell co-stimulatory molecule involved in antigen processing that is also unexpectedly expressed on podocytes in certain experimental and clinical disease states. Podocyte CD80 activation through Toll-like receptor (TLR) 3 and 4 by LPS, independent of T cells, causes proteinuria and foot process effacement ^49^.

Urinary CD80 levels are increased in MCD during relapse but are not increased in FSGS patients or MCD patients in remission ^50^. Sera from MCD patients in relapse, but not in remission, stimulated CD80 expression in cultured podocytes ^51^. The factor(s) in patients’ serum that stimulates podocytes is unknown. Most recently, it was reported that no significant up-regulation of podocyte CD80 was detected in MCD and FSGS patients' biopsies compared with controls using different primary antibodies and immunohistochemical assays, suggesting further confirmation is needed with CD80 in MCD ^52^.

### TNF-α

TNF-α is suggested to be one of the circulating factors that exists in patient plasma of post-transplant recurrent FSGS ^53, 54^. The effect on the podocyte was actin cytoskeleton disruption and activation of β3 integrin. In MCD, it has been suggested that TNF-α synthesis in peripheral mononuclear cells from relapse is increased ^55^. Genome-wide DNA methylation analysis was performed in naïve T helper cells both in relapse and in remission of MCD ^20^ and it was found that the promoter region of TNF-α from relapse has a significant reduction in DNA methylation compared to that from remission in the same individuals, indicating predisposition of TNF-α synthesis in relapse in MCD [personal communication, Dr Yasuko Kobayashi].

Summarizing the data, an excess factor or missing/imbalance of factors in relapse plasma could be the primary cause of MCD, and interesting candidates with biological plausibility are Hpx, suPAR, and TNF-α. PAR1 or uPAR and β3 integrin are therefore potentially activated by circulating factors, and VASP-p is in the pathway downstream of PAR1 or integrins. CD80 is a product of podocyte stimulation by circulating factors. Reorganization of actin by Hpx is dependent on nephrin. The structural changes in actin result in foot process effacement and increase of permeability, which is the core feature in the disease.

The circulating factors might be secreted by peripheral blood cells such as T or B cells by mesangial or endothelial cells in a paracrine manner or by the podocyte itself in an autocrine manner.

## Conclusion

Pathogenic gene mutation analysis in familial MCD has started to reveal insights into underlying mechanisms of pathophysiology in the podocyte, such as endocytosis or Rho GTPase, related to permeability.

We have less evidence of circulating factor activity or from genetic disease in MCD compared to FSGS, perhaps because of less disease severity and lower availability of patient samples in MCD. In terms of circulating factor diseases, findings in FSGS can be examined in relation to MCD. A humanized mouse model might give us a good tool to investigate T cell dysfunction directly related to podocytes.

There are several candidates for biomarkers to predict disease activity or steroid response that allow us to choose precise and acceptable treatment for each individual patient while reducing the side effects of long-term treatment.

New components might be inducible targeting of a specific molecule that is involved in the pathogenesis of MCD both for screening and for treatment.

## Abbreviations

MCD, minimal change disease; SSNS, steroid-sensitive nephrotic syndrome; FSGS, focal segmental glomerulosclerosis; SRNS, steroid-resistant nephrotic syndrome; EMP2, epithelial membrane protein 2; LPS, lipopolysaccharide; KANK, kidney ankyrin repeat-containing protein; ARHGDIA, Rho GDP dissociation inhibitor (GDI) alpha; Th0s, naïve T cells; NFRKB, nuclear factor related to kappaB binding protein; Th17, helper T subset 17; Treg, regulatory T cell; PBMC, peripheral mononuclear cell; PAR1, protease activated receptor 1; VASP, vasodilator stimulated phosphoprotein; suPAR, soluble urokinase plasminogen activator receptor; TLR, Toll-like receptor; TNF-α, tumor necrosis factor alpha.
